# Organelle dysfunction upon *asrij* depletion causes aging‐like changes in mouse hematopoietic stem cells

**DOI:** 10.1111/acel.13570

**Published:** 2022-03-15

**Authors:** Saloni Sinha, Alice Sinha, Prathamesh Dongre, Kajal Kamat, Maneesha S. Inamdar

**Affiliations:** ^1^ Jawaharlal Nehru Centre for Advanced Scientific Research Bangalore India

**Keywords:** aging, Asrij/OCIAD1, endosome, homeostasis, HSC, mitochondria, organelle, proteasome

## Abstract

Aging of the blood system is characterized by increased hematopoietic stem cells (HSCs) and myeloid‐biased differentiation leading to higher propensity for hematological malignancies. Unraveling cell‐intrinsic mechanisms regulating HSC aging could aid reversal or slowing of aging. Asrij/OCIAD1 is an evolutionarily conserved regulator of hematopoiesis and governs mitochondrial, endosomal, and proteasomal function in mammalian stem cells. *Asrij* deletion in mice causes loss of HSC quiescence, myeloid skewing, reduced p53 and increased DNA damage, features attributed to aged HSCs. Mechanistically, Asrij controls p53 ubiquitination and degradation and AKT/STAT5 activation. Asrij localizes to endosomes and mitochondria. As decline in organelle structure and function are common hallmarks of aging, we asked whether Asrij regulates organelle function in aged HSCs. We find that chronologically aged wild‐type (WT) HSCs had reduced Asrij levels. Expectedly, young *asrij* KO mice had reduced AcH4K16 levels; however, transcriptome analysis of KO HSCs showed a modest overlap of gene expression with aged WT HSCs. Further, analysis of organelle structure and function in *asrij* KO mice revealed significant changes, namely damaged mitochondria, elevated ROS; impaired endosomal trafficking seen by increased cleaved Notch1, reduced Rab5; and reduced 26S proteasome activity. Pharmacological correction of mitochondrial and proteasome activity in *asrij* KO mice restored HSC and myeloid cell frequencies. Furthermore, lysophosphatidic acid‐induced Asrij upregulation in aged WT mice rescued mitochondrial and proteasome activity and restored HSC frequency. Our results highlight a new role for Asrij in preventing HSC aging by regulating organelle homeostasis and will help decipher organelle dynamics in HSC longevity.

## Graphical abstract

We propose a role for the stem cell protein Asrij/OCIAD1 in maintaining organelle homeostasis during hematopoietic stem cell (HSC) aging. *Asrij* depletion in mice leads to premature aging of HSCs along with mitochondrial, endosomal, and proteasomal dysfunction. Pharmacological restoration of organelle activity in *asrij* knockout mice reverses phenotypes of premature HSC aging. Asrij could serve as a biomarker of aging and help devise strategies to delay aging or rejuvenate aged HSCs.

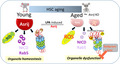

## 1 INTRODUCTION, RESULTS, AND DISCUSSION

Bone marrow hematopoietic stem cells (BM HSCs) constantly combat multiple stressors for blood cell homeostasis. This ability reduces with age leading to functional decline characterized by increased HSCs, myeloid skewing, inflammaging, and clonal hematopoiesis (Mejia‐Ramirez & Florian, 2020). Multiple cell‐intrinsic and cell‐extrinsic factors regulate the genetic and epigenetic landscape, cell polarity, and autophagy to maintain HSCs (Grigoryan et al., 2018; Ho et al., 2017). The dynamic metabolic requirements of HSCs necessitate strict control of mitochondrial (mt) metabolism, endocytic activity, and proteostasis (Gurumurthy et al., 2010; Warr et al., 2013). Low mtROS levels and translation along with rapid proteasome‐mediated protein turnover in HSCs minimize oxidative damage and protein aggregation (Mejia‐Ramirez & Florian, 2020; Hidalgo San Jose et al., 2020). Further, endosomal proteins aid asymmetric localization of cellular components, essential for HSC self‐renewal (Ting et al., 2012). Thus, integrated organelle function is critical to delay HSC aging. Although altered organelle architecture and function are implicated in aging and age‐related diseases (Bouska et al., 2019), very little is known about organelles in HSC aging. Hence, we investigated the role of Asrij, an organelle protein, in HSC aging.

The OCIA (Ovarian Carcinoma Immunoreactive Antigen) domain‐containing protein Asrij/OCIAD1 has a conserved role in post‐translational regulation of signaling to maintain embryonic stem cell potency, and hematopoietic and immune homeostasis. Several mitochondrial, endosomal, and proteasomal components are sensitive to Asrij levels, indicating a possible role for Asrij in organelle homeostasis (Khadilkar et al., 2014, 2017; Kulkarni and Khadilkar et al., 2011; Praveen et al., 2020; Sinha et al., 2013, Sinha, Dwivedi, et al., 2019; Sinha, Ray, et al., 2019). Asrij regulates HSC quiescence and *asrij* deletion in mice triggers HSC expansion, myeloid skewing, DNA damage, and reduced p53 levels (Sinha et al., 2019), phenotypes attributed to an aged hematopoietic system. Hence, we compared organelle homeostasis and HSC aging in control and *asrij* knockout (KO) mice.

**FIGURE 1:**
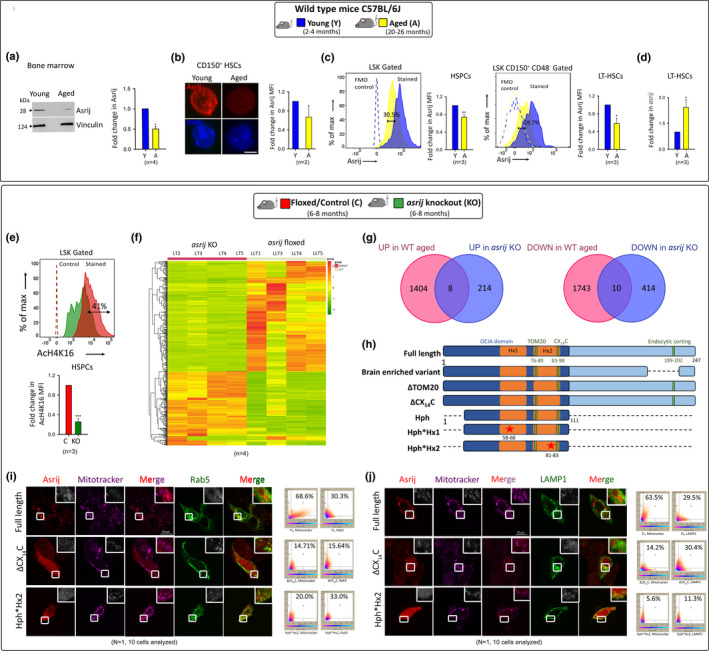
Premature HSC aging‐like changes in *asrij* KO mice. (a) Immunoblot analysis of BM for Asrij levels and graph showing fold change. Vinculin: loading control. (b) Micrographs showing HSCs (LSK CD150^+^) immunostained for Asrij (red). Nuclei marked with DAPI (blue). Scale bar: 2 µm. (c) Representative and summarized flow cytometry data with mean fluorescence intensity (MFI) for Asrij expression in HSPCs and LT‐HSCs. (d) RT‐qPCR for Asrij. (e) AcH4K16 expression in KO HSPCs. Representative flow cytometry data and graph with MFI are shown. (f) Heat map of differentially expressed genes in KO LT‐HSCs. (g) Venn diagrams comparing KO LT‐HSC transcriptome with WT aged dataset (Svendsen et al., 2021). (h) Schematic representation of Asrij constructs. Numbers indicate amino acid positions. Red star shows mutated site. Micrographs of HEK293 cells transfected with Asrij‐FLAG and (i) GFPRab5 or (j) LAMP1‐mGFP construct and stained with Mitotracker Deep Red. Insets show magnified view of the boxed region. Co‐localization plots are to the right of each panel. Error bars denote SEM. *p < 0.05, **p < 0.01 and, ***p < 0.001

Immunoblotting, immunofluorescence, and flow cytometry showed reduced Asrij levels in BM, hematopoietic stem and progenitor cells (HSPCs), and long‐term (LT) HSCs of aged (>20 months) WT mice (C57BL/6J) (Figure 1a–c). Unexpectedly, gene expression analyses showed increased *asrij* transcript in aged WT HSCs (Figure 1d) suggesting complex regulation of expression and possible post‐translational mechanisms that may operate to lower Asrij expression in aged HSCs. Epigenetic and transcriptional changes causally linked with WT HSC aging include reduced histone H4 lysine 16 acetylation (AcH4K16) (Grigoryan et al., 2018) and aberrant gene expression signatures. Flow cytometry of young (6–8 months) KO HSPCs showed reduced AcH4K16 compared to age‐matched controls (Figure 1e), a phenotype reported for aged WT HSCs (Grigoryan et al., 2018), confirming epigenetic dysregulation. However, comparing LT‐HSC transcriptomes of WT aged mice (Svendsen et al., 2021) with that of *asrij* KO mice (see Methods) showed only a modest overlap (Figure 1f,g), suggesting variation in HSC aging signatures.

Asrij harbors multiple motifs that target mitochondria [TOM20 (76–80 aa), CX_14_C (83–98 aa)], endoplasmic reticulum (ER)‐mitochondria contact sites (Cho et al., 2020), endosomes (OCIA domain) (Figure 1h), and proteasome [N‐degron (1–3 aa)]. A naturally occurring brain variant of Asrij lacks an endocytic sorting motif (199–202 aa) but localizes to mitochondria and endosome similar to full length Asrij. Using mutant and/or deletion constructs [∆TOM20, ∆ CX_14_C, Hydrophobic region (Hph), Hph*Hx1 (mutated helix 1), and Hph*Hx2 (mutated Helix 2)] expressed in HEK293 cells, we found that disruption of Hx2 or the CX_14_C reduced Asrij localization to lysosomes and mitochondria (Figure 1i,j; Figure S1a,b). As disrupting organelle targeting motifs in Asrij perturbs its localization, we investigated the effect of *asrij* depletion on organelle homeostasis.

**FIGURE 2:**
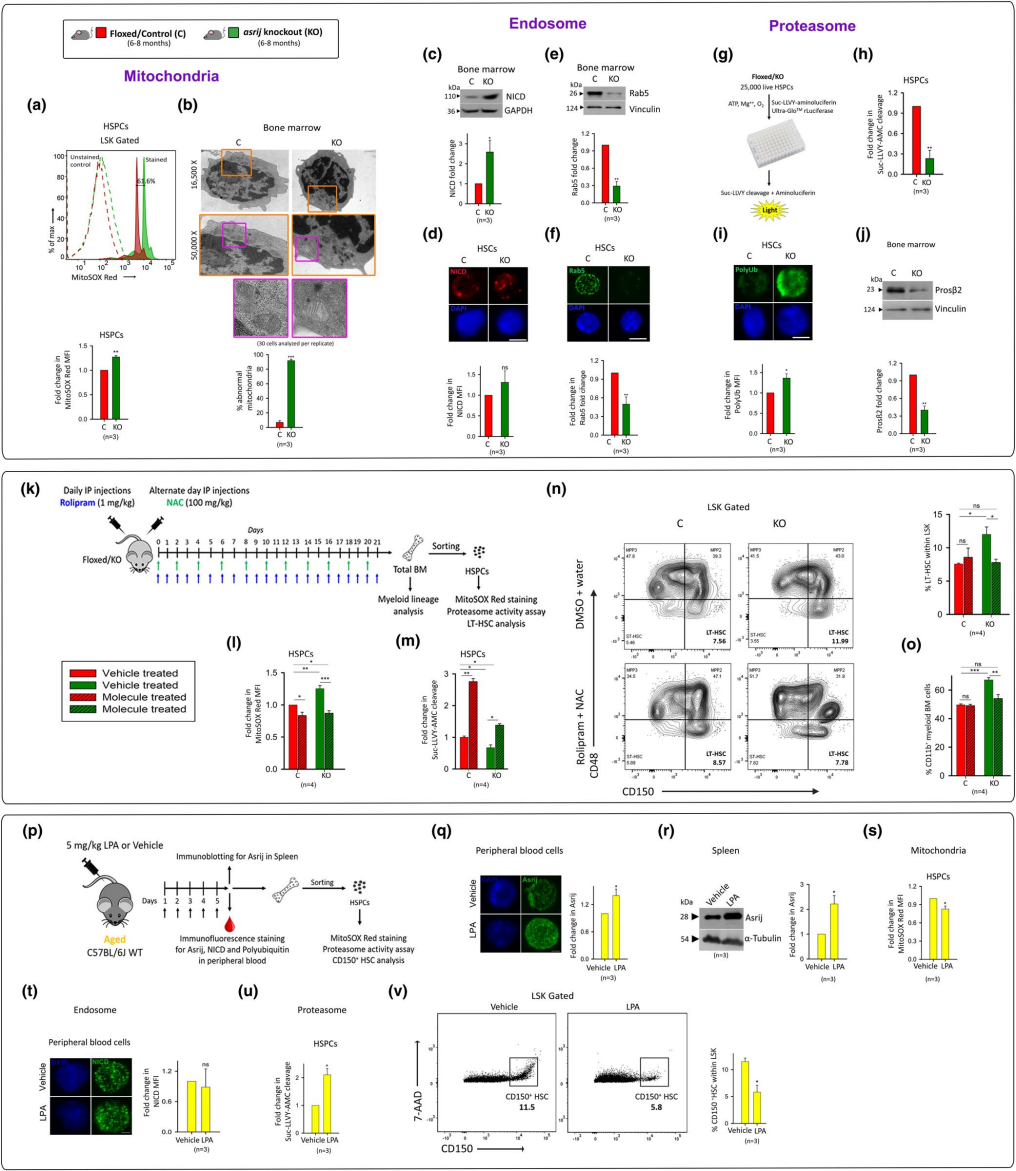
*Asrij* KO HSPCs show organelle dysfunction that can be reversed by pharmacological intervention. (a) Representative and summarized flow cytometry data for mtROS in HSPCs. Graph shows MFI. (b) Representative TEM images and quantification of abnormal mitochondria in BM. (c–h) BM immunoblotting and HSC immunostaining analyses for (c, d) cleaved Notch1 (NICD), (e, f) Rab5, respectively. GAPDH: loading control. Graphs show fold change in protein expression. (g–i) Analysis of proteasome activity in HSPCs. Graph shows fold change in SUC‐LLVY‐AMC cleavage. (j) Immunoblot analysis for Prosβ2 levels in BM. Vinculin: loading control. (k–o) Pharmacological treatment and analysis. (k) Regimen for Rolipram and NAC treatment of mice. Graphs show (l) mtROS and (m) proteasome activity in HSPCs. (n) Representative flow cytometry data and graph showing LT‐HSC percentage within LSK. (o) Percentage of BM CD11b^+^ cells. (p–w) LPA‐mediated upregulation of Asrij in WT aged mice. (p) Regime for LPA treatment. Immunoblot and immunostaining for Asrij in (q) peripheral blood cells and (r) spleen, respectively, (s) mtROS, (t) NICD, and (u) proteasome activity in LPA‐treated cells. (v) Representative flow cytometry data and graph showing CD150^+^HSCs within LSK. Error bars denote SEM. *p < 0.05, **p < 0.01, and ***p < 0.001

OCIAD1/Asrij controls mtComplex I activity and thereby mtROS, in human pluripotent stem cells (Shetty et al., 2018) and also mitochondrial morphology and dynamics (Ray et al., 2021). Flow cytometry showed elevated mtROS levels in young KO HSPCs compared to control (Figure 2a). Further, ultrastructural defects in mitochondrial architecture such as vacuolization and linearization of cristae were seen in KO BM cells (Figure 2b).

*Asrij* null *Drosophila* blood progenitors show stalling of cleaved Notch1 (Notch1 intracellular domain: NICD) in Hrs^+^ endosomes, leading to elevated NICD and ectopic Notch signaling (Kulkarni et al., 2011). We tested whether endocytic transport was similarly affected in mouse KO HSCs. Immunoblotting and immunostaining in KO BM and HSCs showed increased NICD (Figure 2c,d) and decreased Rab5 GTPase (Figure 2e,f) levels. Thus, Asrij is essential for regulated endosomal activity.

Mitochondrial and endosomal machineries crosstalk with the proteasome to ensure cellular quality control (Raimundo & Krisko, 2018). As Asrij plays a conserved role in regulating protein ubiquitination (Khadilkar et al., 2017; Sinha, Dwivedi, et al., 2019; Sinha, Ray, et al., 2019), we reasoned that *asrij* deficiency may affect proteasome. Expectedly, proteasomal activity (Figure 2g–i) and Prosβ2 levels (Figure 2j) were significantly reduced in KO HSPCs and BM, respectively, implying impaired proteostasis, a universal hallmark of aging. Thus, *asrij* depletion causes organelle dysfunction in HSPCs.

To confirm that organelle dysfunction causally leads to HSC aging, we treated *asrij* KO mice with a proteasome activator (Rolipram) and an antioxidant (N‐acetylcysteine) (Figure 2k) and tested for reversal of aging phenotypes. While *ex vivo* single treatment of LT‐HSCs with Rolipram and NAC did not rescue aberrant organelle phenotypes (Figure S1c–e), a combinatorial treatment for 21 days *in vivo* restored organelle activity to near control levels (Figure 2l,m) with reduction in LT‐HSC and myeloid frequencies (Figure 2n,o), thereby attenuating HSC aging. Further, lysophosphatidic acid (LPA)‐induced increase in Asrij in aged WT mice rescued mitochondrial and proteasome activity and restored HSCs to control levels (Figure S2; Figure 2p–v). Thus, we demonstrate that restoring organelle homeostasis by pharmacological intervention can maintain HSC stemness and lineage choice, thereby reversing phenotypes of premature aging in young *asrij* KO HSCs. We propose that Asrij is a critical node in organelle control of HSC aging.

In summary, we provide the first report that HSC aging is associated with Asrij‐dependent simultaneous dysfunction in mitochondrial, endosomal, and proteasomal machineries. Further, we demonstrate that Asrij links organelle function with genetic and epigenetic programs that promote HSC aging and could serve as a biomarker. How Asrij coordinates and contributes to the dynamic interplay among organelles requires further investigation. Realtime analysis of organelle dynamics in young and aged HSCs along with perturbation in regulators such as Asrij could give further insight into the process. A deeper understanding of the organelle‐level regulation of HSC aging could help identify additional aging biomarkers and suggest strategies to rejuvenate aged HSCs or prevent premature HSC aging.

## 2 EXPERIMENTAL PROCEDURES

Please see [Link], [Link].

## CONFLICT OF INTEREST

The authors declare no competing interests.

## AUTHOR CONTRIBUTIONs

MSI and SS conceived the project; SS, AS, PD, and KK performed research and collected, analyzed, and interpreted data; SS and MSI prepared figures and wrote the manuscript.

## Supporting information

App S1

App S2

## Data Availability

RNA‐Seq data have been deposited in the Gene Expression Omnibus (GEO) under the accession number GSE192948. Link for reviewers: https://www.ncbi.nlm.nih.gov/geo/query/acc.cgi?acc=GSE192948. The data that support the findings of this study are available from the corresponding author upon reasonable request.
