# Supplementary material for: Organelle dysfunction upon asrij depletion causes aging‐like changes in mouse hematopoietic stem cells
Source: Aging Cell. 2022 Mar 15;21(4):e13570. doi: 10.1111/acel.13570 (PMC9009118; doi:10.1111/acel.13570)
Supplement: Supplementary file 2 — App S2 [file ACEL-21-e13570-s002.pdf]

Title Page

Organelle dysfunction upon *asrij* depletion causes aging-like changes in mouse hematopoietic stem cells.

Saloni Sinha[1], Alice Sinha[1**], Prathamesh Dongre[1**], Kajal Kamat[1] and Maneesha S. Inamdar[1*]

Inventory of Supplementary Information:

1. Experimental Procedures.
2. Supplementary Figures.
3. Supplementary Figure Legends.
4. Supplementary Table.
5. Supplementary References.

EXPERIMENTAL PROCEDURES

Mice maintenance and genotyping

C57BL/6J was used as the wild type strain. Young (2-4 months) and aged (20-26 months) C57BL/6J mice (Grigoryan et al., 2018; Ho et al., 2017) were bred inhouse and used for the experiments. Homozygous *asrij* floxed and *asrij* whole-body knockout (KO) mice were maintained and genotyped using PCR as described previously (Sinha et al., 2019). Mice of both genders were used and all animal procedures were performed in accordance with the guidelines of the Institutional Animal Ethics committee (IAEC) of JNCASR (Project Number: MSI005).

Isolation of bone marrow (BM) cells

Mouse BM cells flushed from the long bones (femur and tibia) were washed in sterile phosphate buffered saline (PBS) and processed for magnetic/fluorescence activated cell sorting (MACS/FACS) or used for lysate preparation as described previously (Sinha et al., 2019).

Flow cytometry, sorting and antibody staining

Hematopoietic stem and progenitor cells (HSPCs) defined by cell surface markers LSK (Lin^- c-Kit^+ $Sca1^+$) were immunomagnetically (Miltenyi, Germany) sorted and experiments involving flow cytometric analysis of LT-HSCs and myeloid lineage, antibody staining, and gating was performed as described previously (Sinha et al., 2019). Flow cytometry analysis and sorting

was performed on FACS Aria (BD Biosciences, USA) and data obtained was analyzed using FlowJo version 10.6.

RNA-Seq analysis of the *asrij* KO LT-HSC transcriptome

Total RNA was isolated from LT-HSCs (LSK $CD150^{+}$ $CD48^{-}$) sorted from four independent *asrij* floxed (LLT1, LLT3, LLT4 and LLT5) and KO (ArjKOLT2, ArjKOLT3, ArjKOLT4 and ArjKOLT5) mice using RNeasy Mini Kit (QIAGEN, Germany). RNA samples were checked for degradation using Bioanalyzer RNA 6000 Pico kit (Agilent, USA) and quantified using Qubit RNA Assay Kit (Thermo Fisher Scientific, USA). 100 ng of total RNA was used to generate RNA-Seq libraries using NEBNext Ultra RNA Library Prep Kit for Illumina. Libraries were sequenced on the Illumina HiSeq 2500 system (Illumina Inc., USA) using HiSeq Rapid Cluster Kit v2 and HiSeq Rapid SBS Kit v2 (at Clevergene Biocorp Pvt. Ltd., Bangalore, India). Quality of reads was assessed using FastQC. Raw reads were trimmed using Trim Galore to remove Illumina adaptor sequence and low quality reads and then aligned to the GRCm38/mm10 reference transcripts using Bowtie 2. All downstream analyses including principal component analysis, differential expression analysis and statistical tests were performed using custom R scripts. Transcripts with absolute log2 fold change > 1.5 and adjusted p-value < 0.05 were considered differentially expressed. Heat maps were generated using heatmap.2 function from the Bioconductor. To compare *asrij* KO LT-HSC transcriptome with the WT aged LT-HSC dataset, differentially expressed gene lists from Svendsen et al., 2021 were manually compared with *asrij* KO LT-HSC data (Supplementary Table S1) using online tool (https://www.molbiotools.com/listcompare.php) and represented as Venn diagrams. The

RNA-Seq data were deposited at Gene Expression Omnibus (GEO) under the accession number GSE192948.

Generation of tagged expression constructs

Asrij fusion constructs with FLAG or DsRed were generated by introducing desired Asrij fragments upstream of FLAG or DsRed sequences (between SalI and BamHI restriction sites) of plasmids pCMV-Tag2B and pDsRED2-N1 (Clontech, USA). The constructs used were Asrij full-length (1-247 aa), brain enriched variant (Δ185-235 aa), ΔTOM20 (Δ76-80 aa), $\Delta CX_{14}C$ (Δ83-98 aa), Hph (32-111 aa), Hph*Hx1 (58-60 aa changed to Proline), Hph*Hx2 (81-83 aa changed to Proline), GFP-Rab5 and LAMP1-mGFP (both from Addgene, USA).

Cell culture, transfection and co-localization index calculation

2.5×10^4 HEK293 cells maintained in DMEM media containing 10% FBS were transfected after 36 hours with 1 µg of Asrij constructs. After 48 or 72 hours, the transfected cultures were washed with PBS, fixed with 2% paraformaldehyde and processed for immunostaining using standard procedures as described previously (Sinha et al., 2013). For immunostaining, primary antibody was used against FLAG (SIGMA, USA). Co-localization analysis of Asrij with Rab5, LAMP1 and Mitotracker Deep Red was performed using the ZEN image processing software (ZEN, Carl Zeiss Inc) as described previously (Sinha et al., 2018).

MitoSOX Red staining

MitoSOX Red dye (Invitrogen, USA) was used to measure mitochondrial ROS levels. Briefly, immunomagnetically (Miltenyi, Germany) sorted BM HSPCs were incubated with 5 µM MitoSOX Red for 30 minutes at room temperature in dark and washed with PBS. Red fluorescent signal was acquired using flow cytometer in the PE channel and mean fluorescence intensity (MFI) was used for analysis.

Immunofluorescence and confocal microscopy

Immunomagnetically (Miltenyi, Germany) or flow cytometry based sorted BM HSPCs or LT-HSCs were processed for immunostaining using standard procedures as described previously (Sinha et al., 2019). Primary antibodies used were against Asrij (Abcam, USA), CD150, CD48 (both from Invitrogen, USA), Rab5 (BD Biosciences, USA), Notch Intracellular Cleaved Domain 1 (NICD) (CST, USA) and polyubiquitin (Enzo Life Sciences, USA). Secondary antibodies used were coupled to Alexa-Fluor 488 or Alexa-Fluor 568 (Molecular Probes, USA). Images were acquired using Carl Zeiss LSM880 microscope, intensities were measured using ImageJ 1.48v software and adjusted uniformly for brightness/contrast using Adobe Photoshop CS5.

Immunoblotting and densitometry analysis

Immunoblotting of mouse tissues and BM cells was performed as described previously (Sinha et al., 2019). Primary antibodies against Asrij (Abcam, USA), NICD (CST, USA), Prosβ2 (Santa Cruz, USA), and Rab5 (BD Biosciences, USA) were used. Lysates were normalized with respect to loading control GAPDH or Vinculin or α-Tubulin (all from SIGMA, USA). Secondary antibodies used were anti-rabbit or anti-mouse HRP-conjugated (Genei, India). Quantitation

of fold-change in protein levels was done by measuring the band density using ImageJ software and values were normalized to the loading control for plotting. All experiments were performed on three independent biological samples unless stated otherwise.

Real time – quantitative PCR (RT-qPCR)

RNA was extracted from sorted LT-HSCs (LSK $CD150^{+}CD48^{-}$) using RNeasy Mini kit (QIAGEN, Germany). Reverse transcription was performed using 2µg RNA and Superscript II (Thermo Fisher Scientific, USA) according to manufacturer's instructions. The primers used were Asrij-Forward (5'-GTCGACATGTGGAGACTCTTTGCAG-3') and Asrij-Reverse (5'-GGATCCCCAAGGGG GGAGTTTTC-3'). All reactions were performed on three independent biological samples taking duplicates per reaction.

Transmission Electron Microscopy (TEM)

BM cells harvested were fixed using 2.5% glutaraldehyde for 1 hour and then processed to obtain micro-thin sections (1 µm) at the EM facility of Christian Medical College Hospital, Vellore. Cells were imaged at 50,000X magnification on a TEM (Tecnai G2 Spirit 120kV) at IISc, Bangalore. Mitochondria were manually examined for ultrastructural defects such as linearization of cristae and vacuolization and marked abnormal. Imaging and analysis were performed on at least 30 cells from three independent biological replicates.

26S proteasome activity assay

Chymotrypsin-like activity of the proteasome was determined using the Proteasome-Glo™ Chymotrypsin-Like cell-based assay kit (Promega, USA) following manufacturer's instructions. Briefly, 20,000 BM HSPCs were plated per well followed by addition of the substrate and luciferase enzyme in a total reaction volume of 50 µL in a 96-well plate. Following 10 minutes incubation at room temperature, luminescence generated from the hydrolysis of a specific luminogenic proteasome substrate Suc-LLVY aminoluciferin (Succinyl-leucine-leucine-valine-tyrosine-aminoluciferin) and subsequent luciferase consumption of the released aminoluciferin was measured as relative light units (RLU) using Varioskan plate luminometer (Thermo Fischer Scientific, USA). Proteasome activity was plotted as fold change in Suc-LLVY cleavage. Assays were performed in triplicates on three independent biological samples.

Rolipram and N-acetyl cysteine (NAC) treatment of mice

Rolipram (SIGMA, USA) was reconstituted in sterile DMSO (SIGMA, USA) at 5 mg/mL and then diluted in PBS before injection. NAC (SIGMA, USA) was dissolved in sterile water at 30 mg/mL. For rescue of LT-HSC expansion and myeloid skewing, mice were injected intraperitoneally with 1 mg/kg Rolipram or vehicle (DMSO) every day and administered 100 mg/kg NAC or vehicle (water) every alternate day for 3 weeks. Post treatment, mice were sacrificed and BM HSPCs were isolated for proteasome activity assay and MitoSOX Red staining to validate the treatment. Analysis of BM myeloid lineage and LT-HSC frequency was performed as described previously (Sinha et al., 2019).

***Ex vivo* LT-HSC culture with NAC and Rolipram**

Freshly sorted LT-HSCs (LSK $CD150^+$ $CD48^-$) were seeded on fibronectin (SIGMA, USA) coated 8-well chamber slides (Thermo Scientific, Nunc, Lab-Tek) and cultured for 72 hours in Ham's F-12 medium supplemented with 1% Insulin-Transferrin-Selenium, 1% Glutamax, 10mM HEPES (all from Invitrogen, USA), 10 ng/mL human Stem Cell Factor, 10 ng/mL human Thrombopoietin, 0.1% Bovine Serum Albumin (all from SIGMA, USA) and 1X Antibiotic-Antimycotic (Invitrogen, USA) at 37˚ C with 5% CO_2 (modified and adapted from Wilkinson et al., 2019).

LPA treatment of mice

Oleoyl-L-α-lysophosphatidic acid sodium salt (LPA) (SIGMA, USA) was reconstituted in solvent mixture of Chloroform:Methanol:Acetic acid (95:5:5) at 10 mg/mL and then diluted in PBS before injection. To standardize the dose and duration of LPA treatment, WT young mice were injected intraperitoneally with 3 or 5 mg/kg LPA or vehicle (Chloroform:Methanol:Acetic acid) every day for 5 days and blood was collected after 3 and 5 days by retro-orbital bleeding. Post RBC lysis, peripheral blood cells were immunostained for Asrij and tissues were used for immunoblotting. To test for the reversal of organelle dysfunction and HSC aging phenotypes, WT aged mice were injected intraperitoneally with 5 mg/kg LPA or vehicle every day for 5 days. Post treatment, mice were sacrificed and BM HSPCs were isolated for proteasome activity assay and MitoSOX Red staining. Analysis of BM myeloid lineage and LT-HSC frequency was performed as described previously (Sinha et al., 2019).

Statistical analysis and quantification

Statistically significant differences were determined using ANOVA: single factor analysis. Error bars denote standard error of mean. * $p<0.05$, ** $p<0.01$ and *** $p<0.001$ are indicated.

SUPPLEMENTARY FIGURES

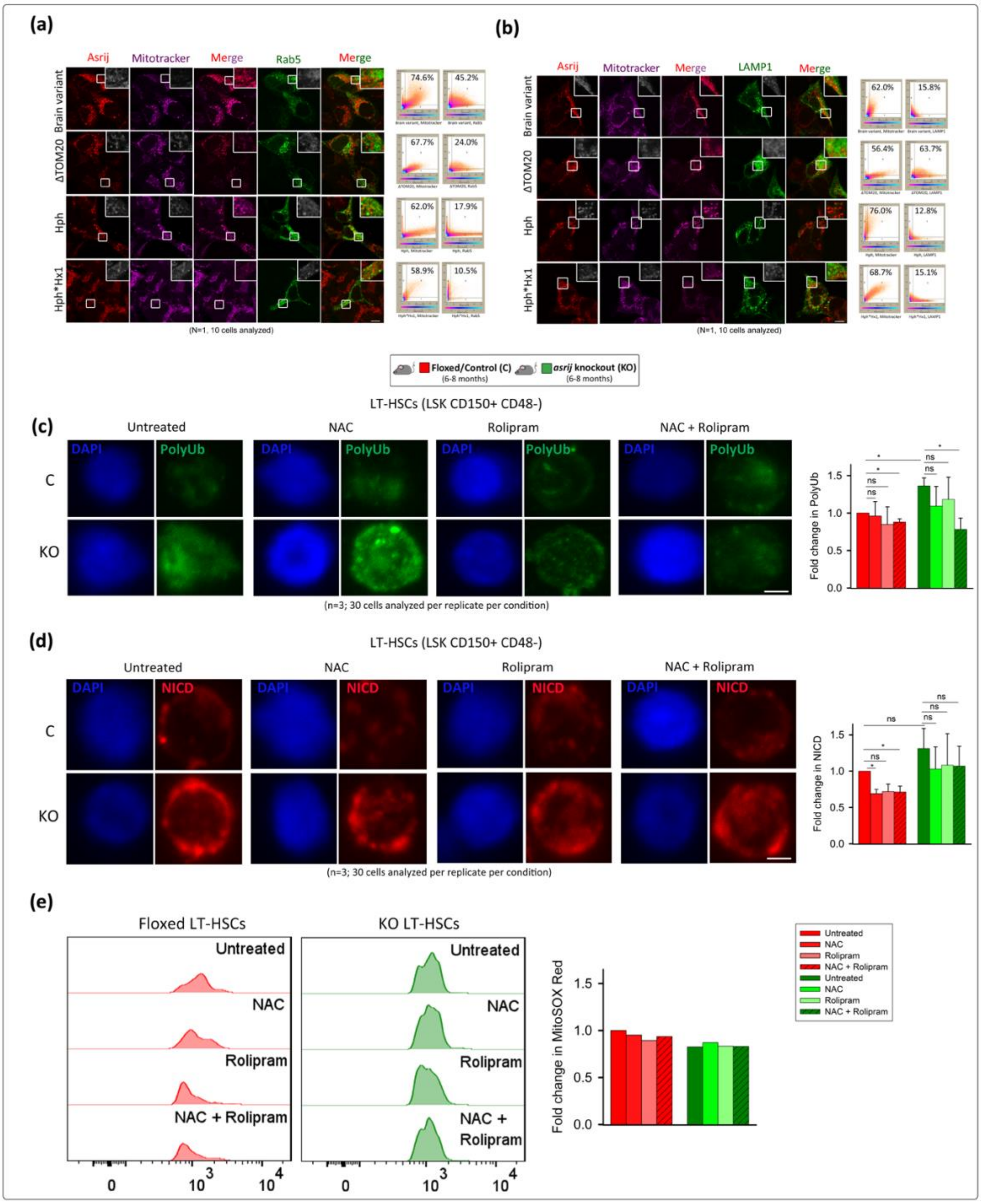

Supplementary Figure S1

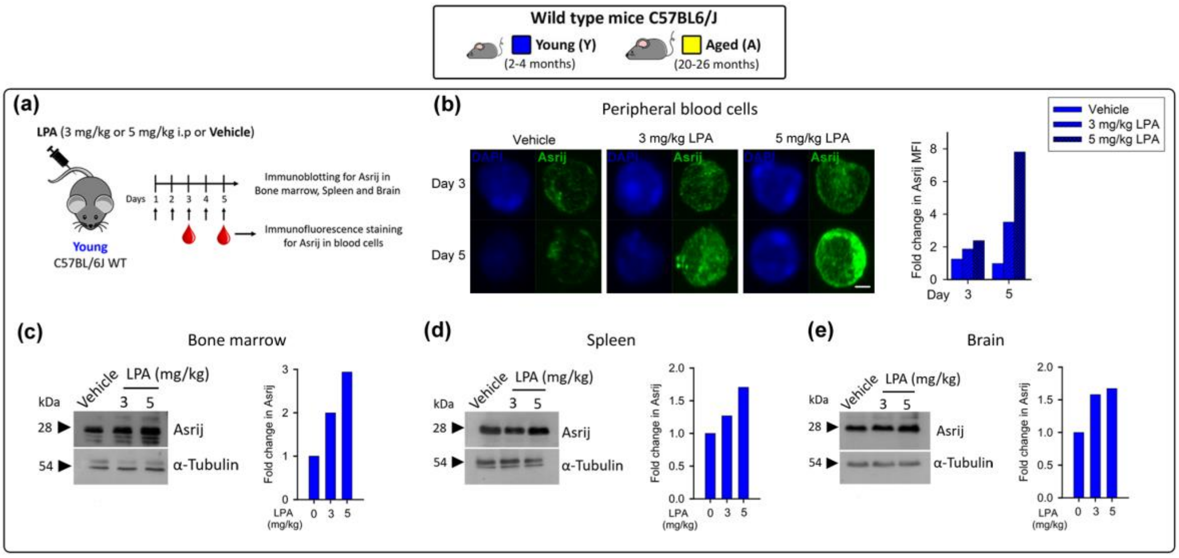

Supplementary Figure S2

SUPPLEMENTARY FIGURE LEGENDS

Supplementary Figure S1. Mapping organelle targeting motifs in Asrij and *ex vivo* pharmacological intervention of *asrij* KO LT-HSCs to rescue organelle defects. **(a, b)** Micrographs of HEK293 cells transfected with Asrij mutant constructs and (a) GFP-Rab5 or (b) LAMP1-mGFP construct, and stained with Mitotracker Deep Red. Insets show magnified view of the boxed region. Co-localization plots are to the right of each panel. Scale bar: 10 µm. Immunostaining for **(c)** polyubiquitin (green) and **(d)** NICD in LT-HSCs post treatment. Nuclei marked by DAPI (blue). Scale bar: 2 µm. **(e)** MitoSOX Red staining analysis post treatment. Error bars denote SEM. ns – non-significant and *p<0.05.

Supplementary Figure S2. Standardization of regime for LPA treatment of mice to upregulate Asrij. (a) Workflow for standardization of dose and duration of LPA treatment for Asrij induction. **(b)** Micrographs showing peripheral blood cells immunostained for Asrij (green). Nuclei marked with DAPI (blue). Scale bar: 2 µm. Graph shows fold change in Asrij MFI. Immunoblot analysis for Asrij in **(c)** BM, **(d)** spleen and **(e)** brain. α-Tubulin: loading control. Graphs show fold change in Asrij.

SUPPLEMENTARY TABLE

Supplementary Table S1. List of differentially expressed genes in *asrij* KO LT-HSCs.

SUPPLEMENTARY REFERENCES

Grigoryan, A., Guidi, N., Senger, K., Liehr, T., Soller, K., Marka, G., . . . Florian, M. C. (2018). LaminA/C regulates epigenetic and chromatin architecture changes upon aging of hematopoietic stem cells. *Genome Biol, 19*(1), 189. doi: 10.1186/s13059-018-1557-3

Ho, T. T., Warr, M. R., Adelman, E. R., Lansinger, O. M., Flach, J., Verovskaya, E. V., . . . Passegue, E. (2017). Autophagy maintains the metabolism and function of young and old stem cells. *Nature, 543*(7644), 205-210. doi: 10.1038/nature21388

Sinha, A., Khadilkar, R. J., S, V. K., Roychowdhury Sinha, A., & Inamdar, M. S. (2013). Conserved regulation of the Jak/STAT pathway by the endosomal protein asrij maintains stem cell potency. *Cell Rep, 4*(4), 649-658. doi: 10.1016/j.celrep.2013.07.029

Sinha, S., Bheemsetty, V.A. & Inamdar, M.S. A double helical motif in OCIAD2 is essential for its localization, interactions and STAT3 activation. *Sci Rep* **8,** 7362 (2018). https://doi.org/10.1038/s41598-018-25667-3

Sinha, S., Dwivedi, T. R., Yengkhom, R., Bheemsetty, V. A., Abe, T., Kiyonari, H., . . . Inamdar, M. S. (2019). Asrij/OCIAD1 suppresses CSN5-mediated p53 degradation and maintains mouse hematopoietic stem cell quiescence. *Blood, 133*(22), 2385-2400. doi: 10.1182/blood.2019000530

Svendsen, A. F., Yang, D., Kim, K. M., Lazare, S. S., Skinder, N., Zwart, E., . . . Bystrykh, L. V. (2021). A comprehensive transcriptome signature of murine hematopoietic stem cell aging. *Blood*. doi: 10.1182/blood.2020009729

Wilkinson, A. C., Ishida, R., Kikuchi, M., Sudo, K., Morita, M., Crisostomo, R. V., Yamamoto, R., Loh, K. M., Nakamura, Y., Watanabe, M., Nakauchi, H., & Yamazaki, S. (2019). Long-term ex vivo haematopoietic-stem-cell expansion allows nonconditioned transplantation. *Nature*, *571*(7763), 117–121. https://doi.org/10.1038/s41586-019-1244-x
